# *PtrA/NINV*, an alkaline/neutral invertase gene of *Poncirus trifoliata*, confers enhanced tolerance to multiple abiotic stresses by modulating ROS levels and maintaining photosynthetic efficiency

**DOI:** 10.1186/s12870-016-0761-0

**Published:** 2016-03-29

**Authors:** Bachar Dahro, Fei Wang, Ting Peng, Ji-Hong Liu

**Affiliations:** Key Laboratory of Horticultural Plant Biology (MOE), College of Horticulture and Forestry Science, Huazhong Agricultural University, Wuhan, 430070 China; Department of Horticulture, Faculty of Agriculture, Tishreen University, Lattakia, Syria

**Keywords:** *Poncirus trifoliata*, Abiotic stress, Alkaline/neutral invertase, Photosynthetic efficiency, Sucrose metabolism, ROS homeostasis

## Abstract

**Background:**

Alkaline/neutral invertase (A/N-INV), an enzyme that hydrolyzes sucrose irreversibly into glucose and fructose, is essential for normal plant growth,development, and stress tolerance. However, the physiological and/or molecular mechanism underpinning the role of A/N-INV in abiotic stress tolerance is poorly understood.

**Results:**

In this report, an A/N-INV gene (*PtrA/NINV)* was isolated from *Poncirus trifoliata*, a cold-hardy relative of citrus, and functionally characterized. *PtrA/NINV* expression levels were induced by cold, salt, dehydration, sucrose, and ABA, but decreased by glucose. *PtrA/NINV* was found to localize in both chloroplasts and mitochondria. Overexpression of *PtrA/NINV* conferred enhanced tolerance to multiple stresses, including cold, high salinity, and drought, as supported by lower levels of reactive oxygen species (ROS), reduced oxidative damages, decreased water loss rate, and increased photosynthesis efficiency, relative to wild-type (WT). The transgenic plants exhibited higher A/N-INV activity and greater reducing sugar content under normal and stress conditions.

**Conclusions:**

*PtrA/NINV* is an important gene implicated in sucrose decomposition, and plays a positive role in abiotic stress tolerance by promoting osmotic adjustment, ROS detoxification and photosynthesis efficiency. Thus, *PtrA/NINV* has great potential to be used in transgenic breeding for improvement of stress tolerance.

**Electronic supplementary material:**

The online version of this article (doi:10.1186/s12870-016-0761-0) contains supplementary material, which is available to authorized users.

## Background

Low temperature, salinity, and drought are major abiotic stresses that significantly inhibit the growth and development of plants and limit the productivity of crops [1]. These stresses perturb cell membranes and protein structures by reducing the availability of water to plant cells [2, 3]. Additionally, these environmental challenges induce oxidative damage in plants by disrupting the delicate balance between production and scavenging of reactive oxygen species (ROS) [4]. Plants continuously suffer from the changing of environmental cues because they are sessile organisms. Thus, they evolved a multitude of adaptive mechanisms to tolerate abiotic stress [5]. Plants have established sophisticated signal transduction pathways to perceive stress signals. The production of second messengers, such as ROS, inositol phosphates, and Ca^2+^, initiate a range of signalling cascades [1]. Consequently, the transcriptome is reprogrammed and a spectrum of protective products are synthesized. These products, along with defensive proteins, function directly or indirectly to protect plant cells from the negative effects of abiotic stress [6].

Among the aforementioned products, some function as compatible solutes, also known as osmoprotectants, which help plants tolerate osmotic stress by maintaining water potential, thereby protecting cellular organelles and essential proteins without interfering with plant metabolisms [7]. Soluble sugars are important osmoprotectants that play a major role in cellular osmotic adjustment by protecting cellular structures exposed to environmental stress [8–11]. Recently, some sugars were proposed to perform a critical role in abiotic stress tolerance by interacting with lipid membranes [8]. On the other hand, emerging evidence points to the role of soluble sugars in ROS scavenging under both biotic and abiotic stresses [12]. Therefore, it is conceivable that particular manipulations of genes involved in sugar metabolism may modulate sugar levels and thus orchestrate stress tolerance in the transgenic plants.

Sucrose (Suc) is one of the predominant products of photosynthesis [13]. Suc is primarily synthesized from Calvin-cycle via sequential action of Suc phosphate synthase (SPS; EC 2.4.1.14) and Suc phosphate phosphatase (SPP; EC 3.1.3.24) [14]. Apart from biosynthesis, Suc is reversibly catabolized by Suc synthase (SS; EC 2.4.1.13) yielding UDP-glucose and fructose, or irreversibly catabolized by invertase (EC 3.2.1.26) yielding glucose (Glc) and fructose (Fru) [15, 16]. In the last decades, Suc and its hydrolytic products, Glc and Fru, were found to serve as signaling molecules that induce the biosynthesis of other osmoprotective substances in response to unfavorable conditions, and influence plant growth and development [3, 8, 17]. Moreover, the integration of invertase-mediated Suc catabolism and signaling mechanism activated by phytohormone regulate the hexokinase-related stress response [14, 18, 19]. Thus, invertase is a key enzyme for plant development and stress response.

There are two major groups of plant invertases (INVs), acidic invertase and alkaline/neutral invertase (A/N-INV). The acidic invertases are further classified into vacuolar (V-INV) and cell wall bound (CW-INV) INV that belong to glycoside hydrolase family 32 (GH32), while A/N-INVs belong to glycoside hydrolase family 100 (GH100) [19, 20]. Both V-INV and A/N-INV are soluble with an acidic isoelectric point (pI), while CW-INV is insoluble with a basic pI [17]. In comparison with CW-INVs and V-INVs, less information is available concerning the functional characterization of plant A/N-INVs [21]. However, emerging evidence has shed light on the potential importance of A/N-INVs in plant development and in the response to biotic and abiotic stress in various plant species, such as *Arabidopsis thaliana* [9, 18, 22, 23]*, Oryza sativa* [24, 25], *Lotus japonicus* [21, 26], and *Triticum aestivum* [20, 27]*.* For instance, the control of cellular hexose concentration by *Arabidopsis thaliana* AtCYT-INV1 was vital for plant development and osmotic stress-induced inhibition of lateral root growth [9]. Moreover, Vargas *et al.* [27] has revealed that the wheat Ta-A-Inv activity was associated with efficient cytosolic Suc hydrolysis during stress conditions. In a very recent study, wheat Ta-A/N-Inv1 was shown to act as a negative regulator of disease resistance by increasing the accumulation of cytoplasmic hexose and reducing the photosynthetic activity of infected leaves [20]. *Arabidopsis At-A/N-InvC* facilitates the energy demands for growth and development [28]. However, the functions of *A/N-INV* genes in cold tolerance remained poorly understood.

*Poncirus trifoliata* (L.) Raf. is extremely cold hardy when it is fully acclimated. In earlier work, we obtained a gene encoding an A/N-INV and several other cold-responsive genes from this plant using suppression subtractive hybridization (SSH) screening [29]. However, we do not know whether this gene, designated as *PtrA/NINV*, contributes to stress tolerance. To test this idea, we first analyzed the expression pattern of *PtrA/NINV* in *P. trifoliata* in response to various abiotic stresses, including cold, salt, and drought stress and in response to ABA, Suc, and Glc treatments. We also examined the subcellular distribution of PtrA/NINV. In addition, we generated transgenic plants overexpressing *PtrA/NINV* to test whether *PtrA/NINV* contributes to abiotic stress tolerance.

## Results

### Identification and sequence analysis of *PtrA/NINV*

We found a cold-induced EST (F2F5) from an SSH screening of a trifoliate orange cDNA library [29]. The EST sequence was used as a query for a BLAST search at NCBI, and it displayed the highest sequence identity (96 %) to a *Citrus clementina* gene (GenBank accession No. XM_006419242.1). As F2F5 is only a partial fragment, we performed RT-PCR with a pair of primers designed based on the sequence of XM_006419242.1 to amplify the full-length sequence, yielding a PCR product of 2037 in length. Sequence analysis demonstrated that it was a full-length sequence with a complete open reading frame (ORF), which encodes a protein of 678 amino acid residues with a predicted molecular weight of 76.4 kDa and a theoretical pI of 6.59. The sequence was named *PtrA/NINV* (*Poncirustrifoliata* A/N-INV). The sequence of F2F5 and the corresponding part of *PtrA/NINV* are identical. Gene structure analysis of *PtrA/NINV* showed that it consists of six exons and five introns (Additional file 1: Figure S1A).

**Fig. 1:**
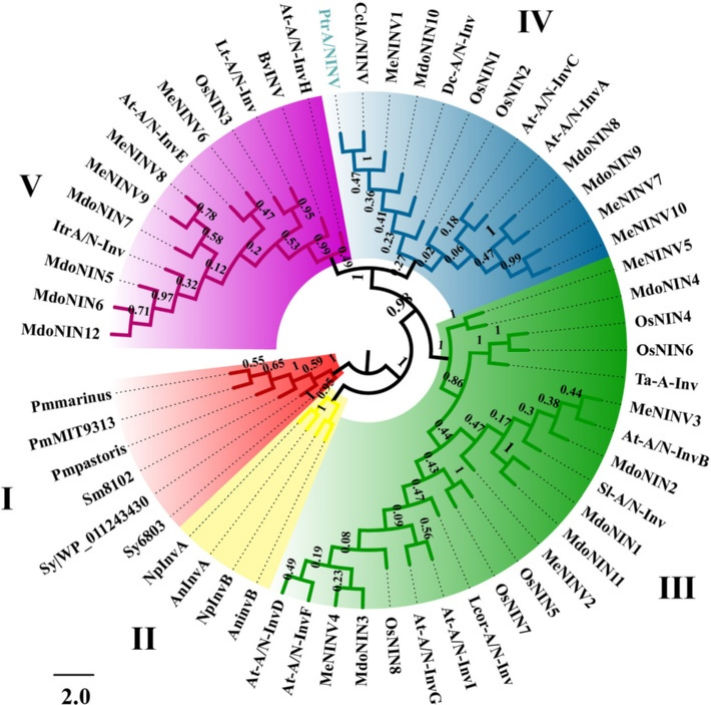
Phylogenetic relationship between *PtrA/NINV* (labeled in blue), and A/N-INVs from other organisms. Groups I and II are unicellular and filamentous cyanobacteria A/N-INVs, respectively, while the remaining groups are from higher plants. In addition to *PtrA/NINV*, 57 amino acid sequences from other organisms are involved, including *Citrus clementina* (*CclA/NINV* ), *Arabidopsis thaliana* (*At-A/N-Inv A-I*), *Beta vulgaris* (*BvINV*), *Daucus carota* (*Dc-A/N-Inv*), *Lolium temulentum* (*Lt-A/N-Inv*), *Lotus corniculatus* (*Lcor-A/N-Inv*), *Oryza sativa* (*OsNIN1-8*), *Malus domestica* (*MdoNIN1-12*), *Manihot esculenta* (*MeNINV1-10*), *Ipomoea trifida (ItrA/N-Inv), Solanum lycopesicum* (*Sl-A/N-Inv*), *Triticum aestivum* (*Ta-A-Inv*), *Nostoc sp*. PCC 7120 (*AnInvA*, *AnInvB*), *Nostoc punctiforme* (*NpInvA, NpInvB*), *Prochlorococcus marinus* MIT9313 (*PmMIT9313*), *P. marinus* subsp. Pastoris (*Pmpastoris*), *P. marinus* subsp. Marinus (*Pmmarinus*), *Synechococcus marinus* WH8102 (*Sm8102*), *Synechococcus* sp. PCC 6301 (*Sy|WP_011243430*), and *Synechocystis* sp. PCC6803 (*Sy6803*). Gene accession numbers are listed in Additional file 5: Table S3. The numbers beside the branches represent bootstrap values based on 1000 replications, and the relative amount of change along the branches is indicated by scale bar

In order to investigate the phylogenetic relationship of A/N-INV genes, we constructed a dendrogram with amino acid sequences from 57 putative INV proteins from various higher plants and cyanobacteria. The sequences are divided into five clades (Fig. 1). The cyanobacterial INVs clustered into group I (unicellular) and group II (filamentous), whereas the rest three groups (group III, IV, and V) were from higher plants. *PtrA/NINV* clustered in group IV and is closely related to sequences from group V. Analysis of the putative protein sequence from *PtrA/NINV*, three genes from group III, six from group IV, and five from group V demonstrated that nine conserved motifs (motif 1–9) are present in all the examined sequences, and comprised the GH100 conserved domain (Additional file 1: Figure S1B). A multiple sequence alignment showed that *PtrA/NINV* exhibited a 58–81 % sequence identity to the tested sequences (Additional file 2: Figure S2).

### Expression pattern of *PtrA/NINV* under various treatments

**Fig. 2:**
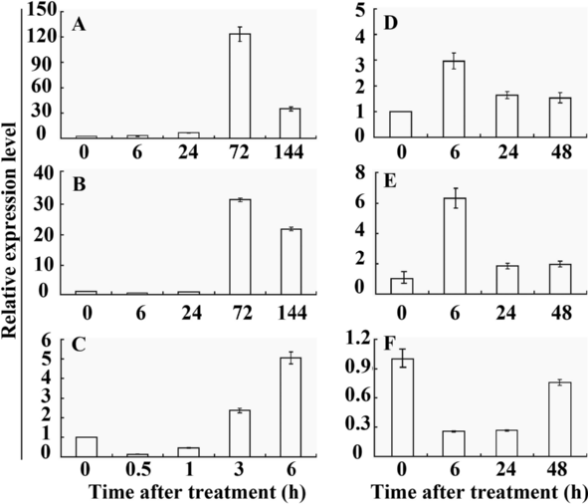
Relative expression pattern of *PtrA/NINV* in *P. trifoliata* under various treatments. Expression of *PtrA/NINV* was analyzed by qRT-PCR using *P. trifoliata* exposed to various treatments, such as 4 °C (**a**), 200 mM NaCl (**b**), dehydration (**c**), 100 μM ABA (**d**), 200 mM sucrose (**e**), and 200 mM glucose (**f**). Transcript level of *PtrA/NINV* at the start of each treatment is set at 1, and those of other time points were accordingly computed. The *Actin* gene was used as an internal control. The values are means ± SE of three biological replicates

A time-course change of *PtrA/NINV* mRNA levels was analyzed by qRT-PCR using *P. trifoliata* seedlings exposed to various treatments, including cold (4 °C), salt, drought, and ABA. Under normal growth conditions, transcript levels of *PtrA/NINV* underwent minor changes (data not shown). By contrast, *PtrA/NINV* was gradually induced within 1 day of cold treatment, but was sharply up-regulated at 3 days to nearly 120 fold of its initial level and then declined at the last day (Fig. 2a). Exposure to salt (200 mM NaCl) for 1 day did not cause a great change in the transcript levels of *PtrA/NINV*, which was elevated by more than 30 fold at 3 days, followed by a decrease at 6 days (Fig. 2b). When the seedlings were treated with dehydration, *PtrA/NINV* mRNA abundance was quickly reduced at 0.5 h, followed by progressive elevation until reaching the peak value at 6 h, which was an approximately five fold increase relative to the initial level (Fig. 2c). We examined the steady-state mRNA levels after an ABA treatment to test whether *PtrA/NINV* is responsive to ABA. As shown in Fig. 2d, the expression level of *PtrA/NINV* was rapidly but transiently increased by ABA treatment at 6 h, followed by a decline. We also determined the expression profiles of *PtrA/NINV* in response to exogenous Suc and Glc treatments. The expression response of *PtrA/NINV* to the Suc treatment was similar to the ABA treatment (Fig. 2e). In contrast, the expression of *PtrA/NINV* was downregulated during the entire Glc treatment (Fig. 2f).

### *PtrA/NINV* localizes in the mitochondria and chloroplast

**Fig. 3:**
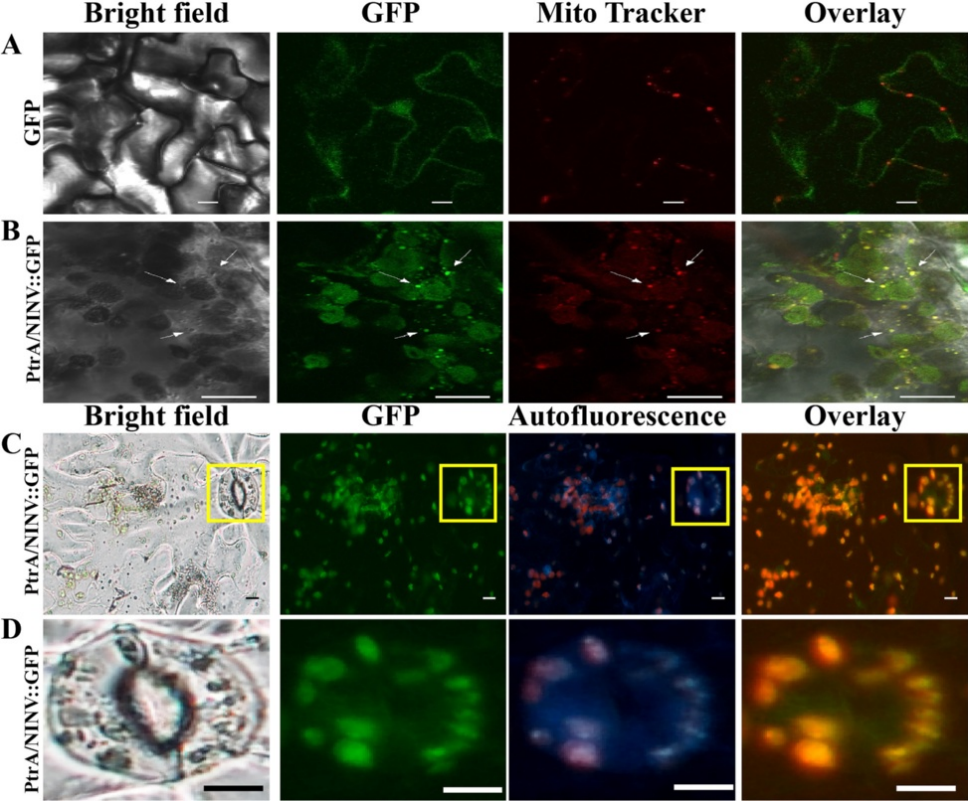
Subcellular localization of *PtrA/NINV* in tobacco epidermal cells. GFP (**a**) or *PtrA/NINV*::GFP (**b**-**c**) was transiently expressed in tobacco epidermal cells and observed under different fields. **d** is a zoom-up of the stomata in the images of **c**. GFP fluorescence was observed using the green channel, and mitochondria were visualized by MitoTracker staining using the red channel. Chlorophyll autofluorescence was visualized using UV channel. The right image of panel **b** is overlaid using the three images on the left. Arrows point to the mitochondria. The right images of (**c** and **d**) were obtained by merging the images of GFP and autofluorescence. Bar =10 μm

We obtained predictions for the subcellular location of *PtrA/NINV* from several dedicated prediction servers [30–34]. This analysis predicted that *PtrA/NINV* accumulates in chloroplasts and/or mitochondria (Additional file 3: Table S1). Interestingly, two initiation sites were identified in the transit peptide sequence (Additional file 3: Table S1). YLoc^+^ [35], a special server that can predict the dual localization of proteins, was also used to predict the localization of *PtrA/NINV*. Based on the whole protein sequence of *PtrA/NINV*, there was a high probability (98.01 %) of chloroplastic localization. Nevertheless, submission of protein sequence from the second initiation site to YLoc^+^ indicated that *PtrA/NINV* could accumulate in both mitochondria and chloroplasts with a probability of 82.23 %. To test these predictions, we transiently expressed *PtrA/NINV::GFP* fusion construct, under the control of the CaMV *35S* promoter, in the epidermis of tobacco leaves. The tobacco leaves expressing GFP gene alone, used as a control, showed a universal distribution of green fluorescence throughout the cells (Fig. 3a). In the case of *PtrA/NINV::GFP* fusion protein*,* confocal laser scanning microscopy revealed presence of green fluorescence in many punctuated particles of 0.5-1 μm in size, which were also labeled with fluorescence from the MitoTracker dye (Fig. 3b). Co-localization of green fluorescence and red color, which is shown in yellow in the merged image, indicated that the protein localized to the mitochondria. We also noticed that the green fluorescence in many large and round structures in the cells, which are possibly chloroplast. To confirm this, we next examined fluorescence of *PtrA/NINV::GFP* fusion protein in the tobacco epidermal cells in absence of MitoTracker. Green fluorescence was clearly observed to co-localized with red autofluorescence of chlorophyll under the UV channel (Fig. 3c-d), implying that *PtrA/NINV* protein also localized to the chloroplasts. Additionally, green fluorescence was not observed in the cytoplasm. These findings indicate that PtrA/NINV is dually targeted to both chloroplasts and mitochondria.

### Generation of *PtrA/NINV*-overexpressing plants

The fact that the expression of *PtrA/NINV* was induced by cold, drought and salt suggests that it may play a role in the tolerance of these abiotic stresses. In order to verify whether this assumption is true, we generated tobacco (*Nicotiana nudicaulis*) transgenic plants overexpressing *PtrA/NINV*, under the control of CAMV *35S* promoter. A total of 60 transformants (T_0_ generation) were identified as positive lines by genomic PCR analysis. Semi quantitative RT-PCR analysis showed that *PtrA/NINV* was overexpressed in four tested lines (data not shown), from which three lines (#7, #8, and #39) with various degrees of *PtrA/NINV* overexpression were studied further.

### Enhanced cold tolerance in the transgenic plants

**Fig. 4:**
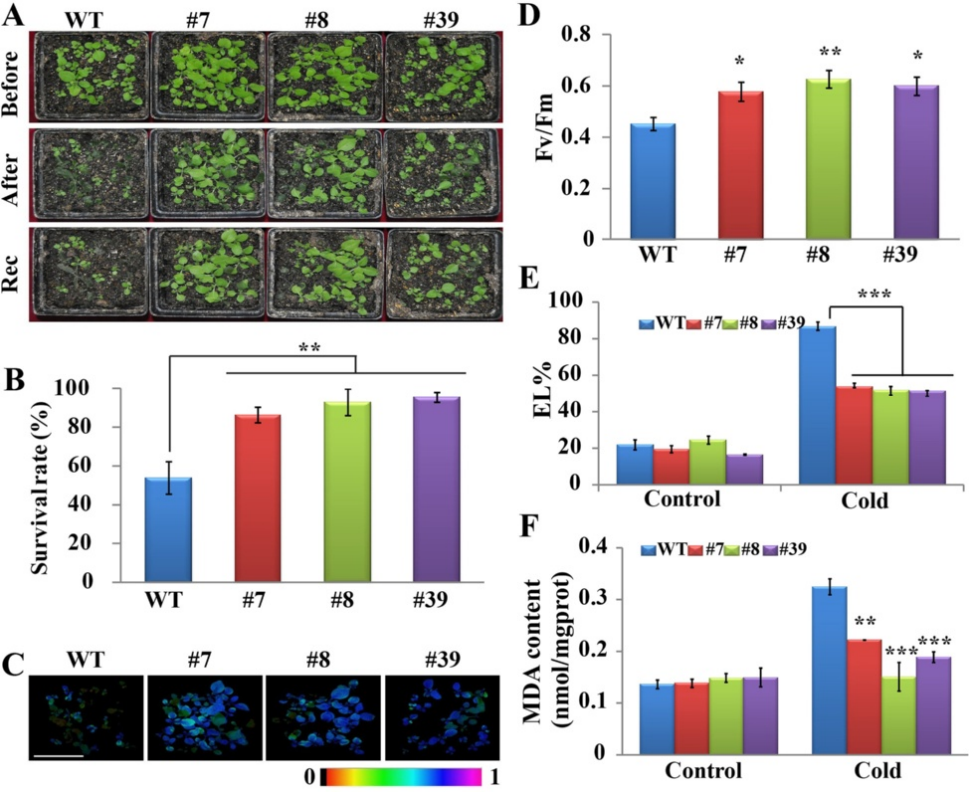
Cold tolerance assay of *PtrA/NINV*-overexpressing transgenic plants. **a** Phenotype of two-week-old plants before and after exposure to −1 °C for 24 h and after recovery at room temperature for 3 days. **b** Survival rate of wild type (WT) and transgenic lines after recovery (*n* = 20). **c**-**d** Chlorophyll fluorescence imaging (**c**) and maximum quantum efficiency of the photochemistry (Fv/Fm, **d**) after the cold treatment of two-week-old plants, which were displayed by Imaging WinGegE software (Walz, Effeltrich, Germany). The false colour scale between 0 and 1 is shown below the imaging. Bar = 5 cm. **e**-**f** Electrolyte leakage (EL, **e**) and MDA (**f**) of 45-days-old plants before and after the cold treatments, which were measured using sampled leaves. Statistically significant differences between the transgenic lines and WT under the same conditions are shown (*P < 0.05, **P < 0.01, ***P < 0.001)

We tested whether the transgenic plants could tolerate low temperature stress by exposing two-week-old transgenic plants (lines #7, #8, and #39) from the third generation and wild type (WT) to 4 °C for 3 days, before they were subjected to a mild stress condition (−1 °C for 1 day). After the cold treatment, the WT seedlings exhibited severe damage and suffered conspicuous water soaking compared to the transgenic plants, which appeared healthy (Fig. 4a). After recovery at room temperature for 3 days, the three transgenic lines grew well and exhibited survival rates ranging from 86.4 to 95.5 %, whereas only 53.8 % of WT resumed growth (Fig. 4b). Chlorophyll fluorescence imaging and the maximum quantum efficiency of photosystem II (Fv/Fm), which are key parameters for the status of photosynthesis [36, 37], were monitored in the plants after the cold treatment. Consistent with the serious damage, the WT seedlings exhibited impaired chlorophyll fluorescence images in comparison with the transgenic lines (Fig. 4c). Meanwhile, the Fv/Fm of the WT plants (0.45) was significantly lower than the Fv/Fm of transgenic plants (0.58–0.63) (Fig. 4d). Electrolyte leakage (EL) and MDA are important markers to assess cell membrane integrity and oxidative damage caused by lipid peroxidation [37]. Under normal condition, the EL and MDA of WT were equivalent to the EL and MDA of the transgenic plants. The cold treatment increased the EL and MDA of the tested lines, but the values of these two parameters in WT were significantly higher than in the transgenic lines (Fig. 4e, f). These data demonstrated that overexpressing *PtrA/NINV* conferred enhanced cold tolerance in the transgenic plants.

### Enhanced salt tolerance in the transgenic lines

**Fig. 5:**
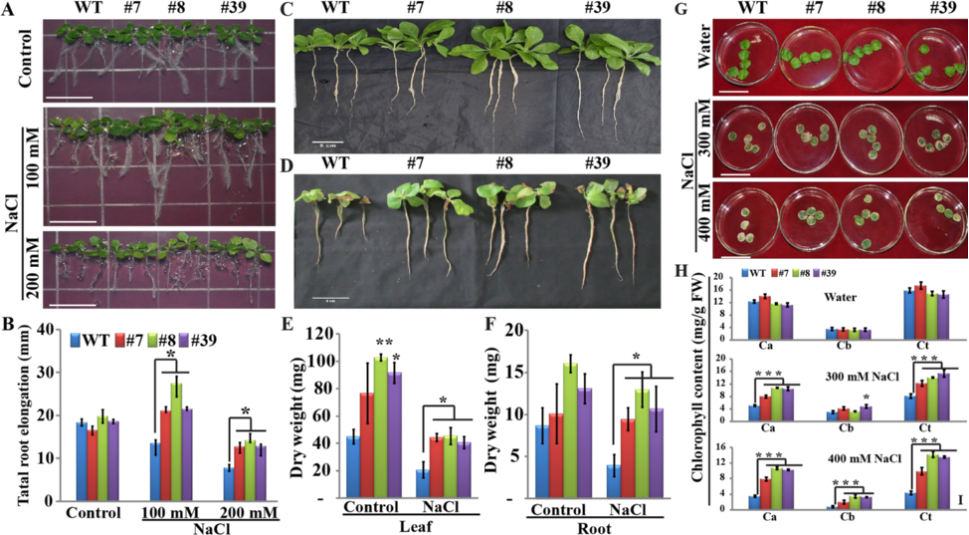
Salt tolerance of *PtrA/NINV*-overexpressing transgenic plants. **a**-**b** Growth performance (**a**) and total root length (**b**) of in vitro seedlings of transgenic lines and wild type (WT) on MS medium without or with salt (100, and 200 mM NaCl) (*n* = 3). **c**, **d** Comparison between one-month-old transgenic lines and WT plants grown in a hydroponic solution without (**c**) or with 150 mM NaCl (**d**). (*n* = 3). **e**-**f** Dry weight of leaves (**e**) and roots (**f**) of hydroponically growing plants. (*n* = 3). **g**-**h** Comparison of leaf disk phenotypes (**g**) and chlorophyll contents (**h**) between transgenic lines and WT immersed into water or salt solutions (300 and 400 mM NaCl) for three days (*n* = 5). Bar = 1 cm (**a**, **b**) or 5 cm (**c**, **d**, **g**). Statistically significant differences between the transgenic lines and WT under the same conditions are shown (*P < 0.05, **P < 0.01, ***P < 0.001)

The induction of *PtrA/NINV* by salt stress promoted us to evaluate the capacity of the transgenic plants to tolerate salt stress. We first checked growth performance of one-week-old *in vitro* seedlings on MS medium added with or without salt. In the absence of salt, we did not observe a difference in plant phenotype and root length between transgenic lines and WT. In contrast, when the seedlings grew on media containing NaCl (100 and 200 mM) for one week, the total root elongation of WT was significantly inhibited relative to the three transgenic lines (Fig. 5a, b). We also used hydroponic culture to test the salt stress tolerance of the transgenic plants. When the seedlings grew for 2 weeks in the hydroponic solution without salt, the transgenic plants were larger than the WT (Fig. 5c). Adding 150 mM NaCl to the hydroponic solution retarded the growth of all the tested lines, but the growth of WT was evidently suppressed more seriously (Fig. 5d). Measurement of biomass based on dry weight of the leaves and roots indicated that the transgenic lines grew better than WT (Fig. 5e-f).

Next, we checked salt stress tolerance by immersing leaf discs prepared from one-month-old plants for 3 days in water or in higher concentrations of salt solution (300 and 400 mM NaCl). We did not detect differences when they were incubated in water (Fig. 5g, h). By contrast, in the presence of 300 or 400 mM NaCl, the leaf discs from WT exhibited more serious bleaching and decreased chlorophyll contents when compared with those of the transgenic lines (Fig. 5g, h).

**Fig. 6:**
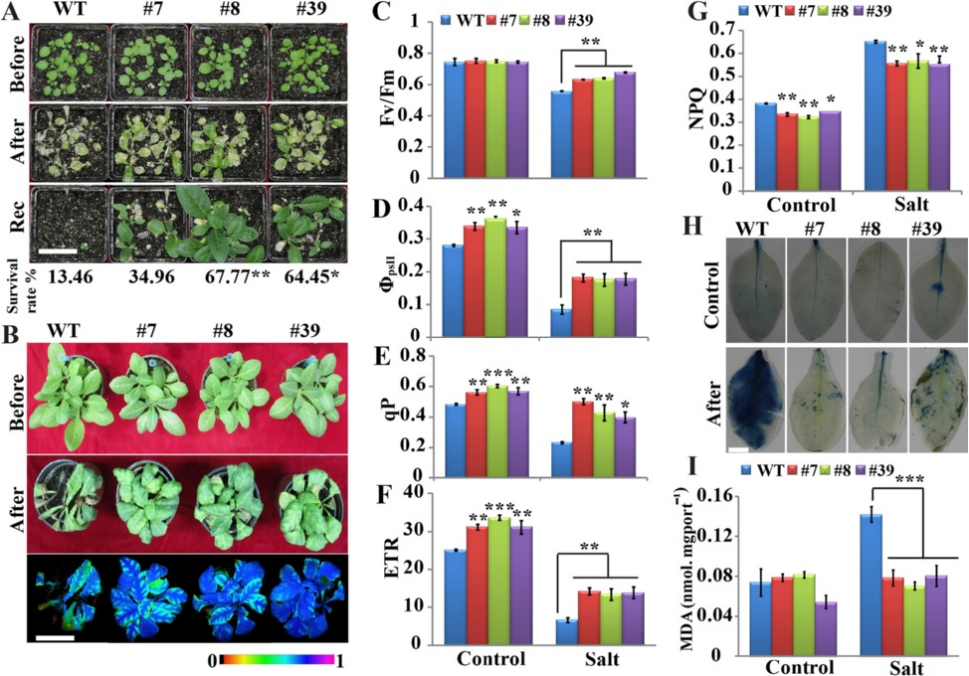
Salt stress tolerance assay of potted plants. **a** Growth phenotype of two-week-old transgenic lines and wild type (WT) before and after spraying with 300 mM NaCl and after a two-week recovery period. Survival rates of each line are shown below the bottom panel (*n* = 12). **b** Treatment of potted plants with 300 mM NaCl for 45 days (*n* = 4), and observation of chlorophyll fluorescence imaging. False colour scale between 0 and 1 is shown below the imaging. **c**-**g** Analysis of photosynthesis-related parameters, including Fv/Fm (**c**), the operating quantum efficiency of the photochemistry (Φ_PSII_, **d**), photochemical quenching (qP, **e**), non-photochemical quenching (NPQ, **g**), and electron transfer rate (ETR, **f**), which were recorded using the dark–light induction curve, and displayed using the Imaging WinGegE software (Walz, Effeltrich, Germany). **h**-**i** Evans blue staining (**h**) and MDA level (**i**) before and after salt stress (*n* = 3). Bar = 5 cm (**a**, **b**) or 1 cm (**h**). Each experiment was repeated at least twice. Statistically significant differences between the transgenic lines and WT under the same conditions were shown (*P < 0.05, **P < 0.01, ***P < 0.001)

Finally, we used potted plants to investigate the salt tolerance of transgenic plants. When 15-days-old plants were sprayed with a salt solution (300 mM NaCl) for two weeks, followed by recovery for 15 days, the transgenic lines showed better growth after the imposition and relief of the salt stress (Fig. 6a). Survival rates of the transgenic lines ranged from 35 to 68 %, whereas only 13.5 % of WT plants survived after recovery. We used different methods to assess whether salt differentially affected photosynthesis in WT and the transgenic lines. We treated 45-days-old plants with 300 mM NaCl for 45 days and checked chlorophyll fluorescence using false color imaging. Following exposure to the salt stress, the transgenic lines demonstrated better fluorescence than did the WT, implying that they contained higher chlorophyll contents (Fig. 6b). Although the Fv/Fm that we observed in untreated WT and transgenic plants were equivalent to each other, WT plants exhibited a greater decrease relative to the transgenic lines when they were subjected to salt stress (Fig. 6c). We then analyzed several other parameters related to photochemical efficiency, including Φ_PSII_, qP, ETR, and NPQ. Of note, the transgenic lines exhibited higher levels of Φ_PSII_, qP, ETR, than did the WT irrespective of salt stress (Fig. 6d-f). By contrast, we observed lower levels of NPQ in the transgenic lines (Fig. 6g). Examination of cell viability and MDA content showed that in the absence of salt stress, cell death was scarcely observed in the tested lines, as shown by Evans blue staining (Fig. 6h). Even though the salt treatment greatly stimulated cell death in the leaves of WT and transgenic lines, the latter was less serious relative to the former. In addition, the transgenic lines had significantly lower MDA levels under salt stress compared to the WT (Fig. 6i). Taken together, all of these data demonstrate that *PtrA/NINV* overexpression greatly improved salt-stress tolerance.

### Overexpression of *PtrA/NINV* improves tolerance to drought stress

**Fig. 7:**
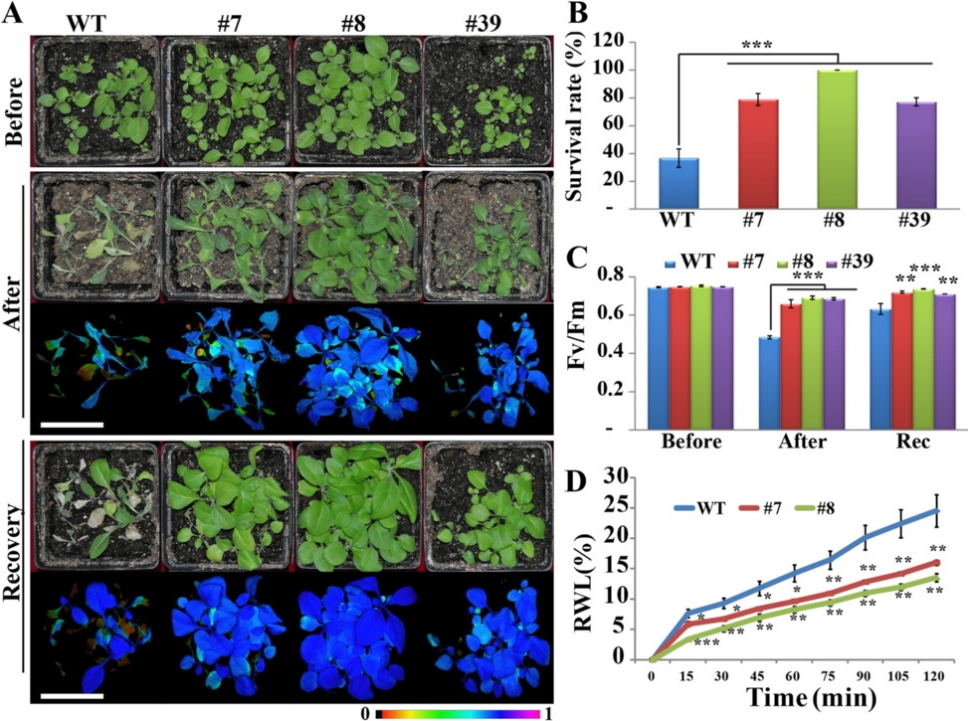
Determination of drought tolerance of *PtrA/NINV-*overexpressing plants. **a** Plant phenotype of transgenic lines and wild type (WT) before and after a two-week water deprivation treatment and after a 3-days-recovery period (*n* = 20). Bar = 5 cm. Chlorophyll fluorescence imaging is shown below the relevant panels, and the false colour scale between 0 and 1 is presented. **b** Survival rates of transgenic lines and WT scored after the recovery. **c** Fv/Fm ratio of transgenic lines and WT before and after drought treatment. **d** Relative water loss rates measured during dehydration of leaves prepared from transgenic lines (#7 and #8) and wild type (*n* = 4). Each experiment was repeated at least twice. Statistically significant differences between the transgenic lines and WT at the same time points are shown (*P < 0.05, **P < 0.01, ***P < 0.001)

As drought is also a major factor causing osmotic stress, we made efforts to test drought tolerance of the transgenic plants. To this end, we withheld water for two weeks from two-week-old plants of WT and transgenic lines, and then returned to regular watering for 3 days to allow for a recovery. After two weeks deprived of irrigation, the WT plants showed visual symptoms of drought-associated phenotypes, such as leaf rolling, wilting and necrosis, whereas the transgenic lines exhibited better growth (Fig. 7a). About 63.3 % of WT plants failed to survive after recovery, whereas 77.0–100 % of the transgenic lines remained healthy and exhibited vigorous growth (Fig. 7b). After the drought stress and at the end of recovery, the transgenic plants displayed better fluorescence imaging relative to WT (Fig. 7a). In parallel, Fv/Fm was significantly lower in WT than in the transgenic plants after drought stress and recovery (Fig. 7c). We tested whether *PtrA/NINV* affects the rate of water loss because the water retention rate is correlated with the magnitude of drought tolerance. To this end, leaves were detached from one-month-old plants of WT and transgenic lines (#7 and #8), and dehydrated in an ambient environment for 2 h. WT leaves displayed pronounced leaf wilting, whereas the transgenic leaves retained better turgor after dehydration (data not shown). Measurement of relative water loss revealed that water loss increased progressively during the course of dehydration in both WT and transgenic lines. However, the WT leaves exhibited quicker and greater water loss in comparison with the transgenic ones. At the last time point, the water loss rate of WT was 24.6 %, while the transgenic leaves lost 13.55–16 % of their water (Fig. 7d). Altogether, these results demonstrated that *PtrA/NINV* overexpression confers enhanced drought tolerance in the transgenic plants.

### *PtrA/NINV-*overexpressing plants accumulate less ROS and contain higher antioxidant enzyme activities

**Fig. 8:**
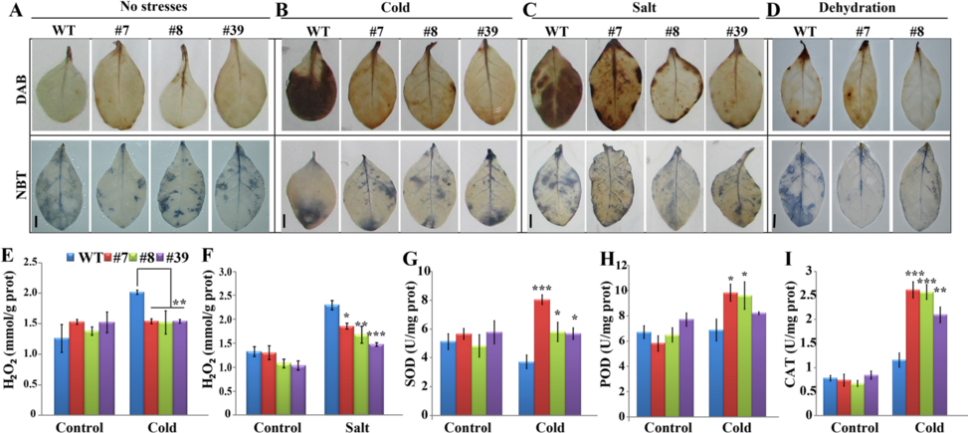
Determination of ROS accumulation and antioxidant enzymes activities. **a**-**d** *In situ* accumulation of H_2_O_2_ and O_2_^•-^ in the transgenic lines and wild type (WT) under normal conditions (**a**), cold (**b**), salt (**c**), and dehydration (**d**), as revealed by staining with DAB and NBT, respectively. Bars = 1 cm. **e**-**f** Quantitative measurement of H_2_O_2_ contents before and under cold (**e**) and salt (**f**) stress. **g**-**i** Enzyme activities of SOD (**g**), POD (**h**), and CAT (**i**) in the transgenic lines and WT before and after cold treatment (*n* = 3). Statistically significant differences between the transgenic lines and WT under the same conditions are shown (*P < 0.05, **P < 0.01, ***P < 0.001)

In the stress tolerance assay, we noticed that the transgenic lines had lower MDA values under the stresses, implying that they suffered from lower degrees of oxidative damages. As ROS is a major factor causing oxidative stress, we assessed the accumulation of ROS, in particular H_2_O_2_ and O_2_^·-^, in WT and the transgenic lines under stress. We used histochemical staining with 3, 3’-diaminobenzidine (DAB) and nitro blue tetrazolium (NBT) to reveal *in situ* production of H_2_O_2_ and O_2_^·-^, respectively. As shown in Fig. 8a, DAB and NBT similarly stained the leaves of WT and transgenic lines under normal growth conditions (Fig. 8a). In the presence of the stressors, the WT exhibited deeper and more intense DAB staining patterns when compared with the transgenic lines, but no difference in DAB staining was detected among the tested lines (Upper panels of Fig. 8b-d). By contrast, no dramatic difference in NBT staining was observed between WT and the transgenic lines following the cold and salt treatments, whereas the transgenic lines were stained to lesser degrees compared to WT following the dehydration treatment (Bottom panels of Fig. 8b-d), implying that the accumulation of O_2_^·-^ in the transgenic lines may be only mitigated under dehydration. In order to verify the histochemical staining, we quantified the levels of H_2_O_2_ in the cold and salt-treated samples using a detection kit. Consistent with the histochemical staining results, quantitative measurement showed that the levels of H_2_O_2_ in the transgenic lines was significantly lower than in WT during the cold and salt treatments, but that no difference was noticed in the absence of the stress treatment (Fig. 8e-f). These results indicated that the accumulation of ROS, in particular H_2_O_2_, was prominently alleviated in the transgenic plants under stress, consistent with the lower levels of oxidative damage in these lines.

The crucial role of antioxidant enzymes in ROS scavenging prompted us to examine the activities of three important enzymes (SOD, CAT, and POD) in WT and the transgenic lines during the cold treatment. We found that in the control conditions there was no significant difference between WT and the transgenic lines, whereas activities of the three enzymes were significantly higher in the transgenic plants compared to WT when the plants experienced cold stress (Fig. 8g-i).

### Transgenic plants show higher A/N-INV activity and reducing sugar levels

**Fig. 9:**
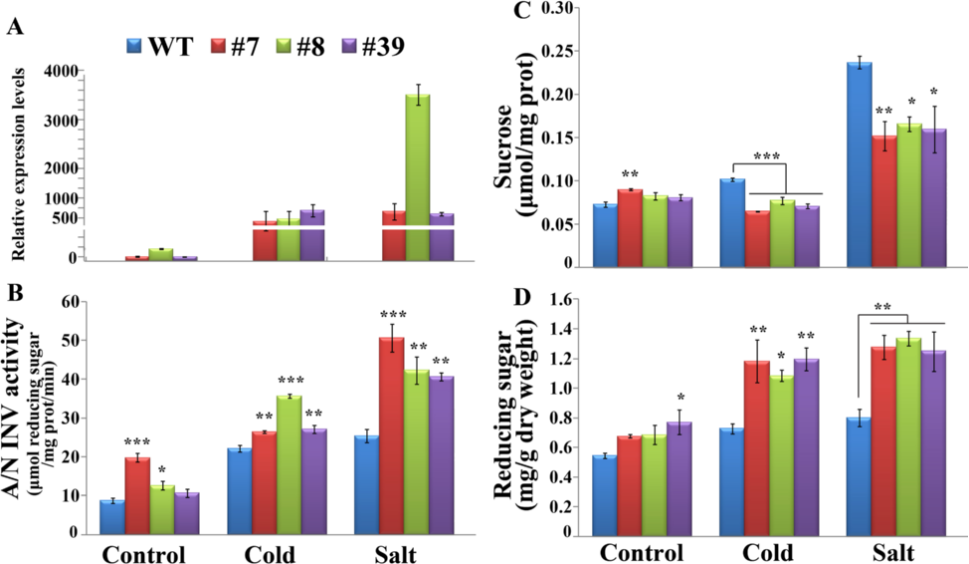
Analysis of *PtrA/NINV* mRNA abundance, A/N-INV activity, and sugar levels. **a**-**d** Transcript levels of *PtrA/NINV* (**a**), A/N-INV activity (**b**), sucrose levels (**c**) and reducing sugar contents (**d**) in the transgenic lines and WT under normal growth conditions and under cold and salt stress (*n* = 3). Statistically significant differences between the transgenic lines and WT grown under the same conditions are shown (*P < 0.05, **P < 0.01, ***P < 0.001)

To investigate how the overexpression of *PtrA/NINV* affects the content of endogenous sugar, we analyzed the activity of A/N-INV, the content of Suc and reducing sugars in WT and the transgenic lines before and after cold or salt treatment. Transcript levels of *PtrA/NINV* in the transgenic plants were dramatically elevated over WT under normal conditions and in response to cold and salt stress (Fig. 9a). In line with the expression patterns, the transgenic plants contained significantly higher A/N-INV activity than WT under both normal conditions and in response to the cold and salt treatments (Fig. 9b). Although the Suc levels in the transgenic lines and WT were comparable under normal growth conditions, the transgenic lines had significantly lower Suc levels when compared with the WT during the cold and salt treatments WT (Fig. 9c). By contrast, during the cold- and salt-stress treatments, the reducing sugar levels of the transgenic plants were significantly higher than in WT (Fig. 9d), concurrent with the decrease of Suc under the same conditions. These results demonstrated that overexpression of *PtrA/NINV* promotes Suc degradation to produce more Glc and Fru under stress conditions.

## Discussion

Soluble sugars are assumed to promote energy demanding processes, including growth, development and stress response through sugar signaling cascades [17]. Some genes involved in sugar metabolism have been analyzed and functionally characterized. However, so far the information on A/N-INV in cold-stress tolerance is limited. Therefore, a functional characterization of the A/N-INV genes will provide a better understanding of the role of Suc metabolism in physiological processes.

In the present study, an A/N-INV gene *PtrA/NINV* was isolated from trifoliate orange. The phylogenic analysis showed that *PtrA/NINV* clustered into clade IV, the members of which were computationally predicted or experimentally proven to localize to mitochondrion [18, 24, 28, 38–40]. Moreover, the dendrogram revealed that *PtrA/NINV* was closely related to group V members that have been reported to localize to chloroplasts [24, 38, 41]. Based on these data, we suggested that *PtrA/NINV* was possibly targeted to both chloroplast and mitochondria. This idea was partially supported by a bioinformatics analysis that utilized diverse tools for the prediction of subcellular localization. Localization of *PtrA/NINV* in both chloroplast and mitochondria was further experimentally verified by transient expression in tobacco epidermal cells, indicating that *PtrA*/*NINV* is truly a dual-targeted protein. Previously, A/N-INVs were believed to accumulate exclusively in the cytoplasm [9, 14]. Later, many reports have demonstrated that this protein accumulates in mitochondria (*OsNIN1*, *At-A/N-InvA*, *At-A/N-InvC*) [18, 24, 28, 42], chloroplasts (*OsNIN3*, *At-A/N-InvE*) [24, 41], or nuclei [23]. To the best of our knowledge, our work represents the first to demonstrate that an A/N-INV protein displays dual targeting to both mitochondrion and chloroplast. It is worth mentioning that dual localization in both mitochondria and plastids has been previously observed in more than 50 proteins, and this phenomenon is assumed to be caused by alternative splicing or translation initiation [43]. For example, alternations in the translation sites of *Arabidopsis AtDEF1* and *AtZn-MP* protein sequences, which contain two in-frame initiation sites, led to changes in the targeting of these proteins to different organelles [44, 45]. We also detected two possible initiation sites at the N-terminus of *PtrA/NINV*, which may be responsible for the dual targeting of this protein. As mitochondria and chloroplasts have many of the same enzymatic pathways due to the dual targeting of proteins [46], it is conceivable that targeting of *PtrA/NINV* in chloroplasts and mitochondria might have been evolutionarily selected to execute similar A/N-INV catalyzed reactions in these two organelles. In this regard, several reports have demonstrated that Suc is present in both plastids and mitochondria [41, 42, 47, 48]. Therefore, it is conceivable that A/N-INV may play a crucial role in Suc metabolism in these two organelles.

*PtrA/NINV* was up-regulated under multiple abiotic stresses, suggesting that it may play a role in tolerance to various abiotic stresses. To verify this assumption, we generated transgenic tobacco plants constitutively expressing *PtrA/NINV* via *Agrobacterium*-mediated transformation. We overexpressed *PtrA/NINV* in the three selected lines before and after stress treatment relative to the WT, which was consistent with the elevation of A/N-INV activities, although the mRNA level and enzyme activity differed in orders of magnitude. These results suggest that *PtrA/NINV* functions normally in the transgenic lines and may participate in the Suc catabolism. This is supported by the prominent reduction in the levels of Suc and a concomitant increase in the levels of reducing sugars in the transgenic plants relative to WT, implying that *PtrA/NINV* may perform a catalytic role in the degradation of Suc into Glc and Fru [16, 49]. Concurrent with the metabolic changes, the transgenic plants displayed enhanced tolerance to abiotic stresses, including high salinity, low temperature, and drought. These data indicate that genetic manipulation of *PtrA/NINV* alters sugar metabolism and corroborates the idea that the elevation of reducing sugars renders the plants more endurable to adverse environmental cues [8, 50, 51].

The water loss induced by various types of abiotic stress may lead to disorders in cellular organelles and the malfunction of membranes [2, 3]. Plants generally accumulate a variety of compatible solutes, also known as osmoprotectants, to overcome the osmotic imbalance induced by abiotic stresses [52]. Soluble sugars are widely accepted as crucial osmoprotectants, as they can function to stabilize cellular membranes by replacing water molecules when plants are exposed to abiotic stress, thereby keeping membrane surfaces hydrated and sustaining the space between phospholipid molecules to prevent membrane damage [3, 53]. In this study, the transgenic lines contained a greater amount of reducing sugars, including Glu and Fru, due to higher A/N-INV activity. This result implied that the transgenic lines hydrolyzed Suc more efficiently than the WT. Suc degradation into glucose and fructose by invertases has been suggested to double the osmotic contribution, which is conducive for sustaining favorable cellular turgor, and thus facilitating water inflow to drive cell expansion in response to different abiotic challenges [17, 27]. In this regard, the transgenic lines may exhibit a better capacity for adjusting the osmotic potential in response to the abiotic stress, rendering them more tolerant to the stress relative to the WT.

ROS is produced in plants under normal growth conditions, but the level is tightly controlled by delicate ROS-scavenging machinery composed of either enzymatic or metabolic antioxidants [54]. However, when plants are exposed to abiotic stress, the accumulation of ROS is largely accelerated, which will lead to oxidative stress if the ROS are not timely detoxified. ROS at high levels is toxic or detrimental to plant cells due to their negative impact on proteins and nucleic acids, leading to lipid peroxidation and membrane damage [4, 5, 12]. Therefore, the magnitude of stress tolerance is, largely if not fully, dependent upon the potential of ROS scavenging. Accumulating evidence demonstrates that reducing sugars can act as ROS scavengers and play a critical role in alleviating the accumulation of ROS, which is a pivotal strategy for plants to cope with abiotic stress [8, 12, 50, 55, 56]. On the other hand, we also noticed that the transgenic lines displayed higher activities of antioxidant enzymes, such as SOD, POD and CAT relative to the WT. Although we did not dissect the cause and effect relationship between increased A/N-IV activity and the antioxidant enzyme activities, such a correlation has been previously reported by other researchers [18, 20, 57]. In keeping with this, the transgenic lines with higher levels of reducing sugars may hold a more powerful system to remove the ROS under the tested stresses compared to WT. Consequently, the transgenic lines seem to suffer from less oxidative stress, suggesting that the damage in the cellular compartments and components could be properly mitigated [58]. This result is consistent with the reduced cell death, lipid peroxidation and better membrane integrity. Therefore, lower ROS accumulation is partly responsible for the enhanced stress tolerance in the transgenic lines overexpressing *PtrA/NINV.*

Chloroplasts are major sources of ROS production in plant cells exposed to adverse environments [4, 5, 50]. Therefore, it is conceivable that this organelle could experience oxidative damage when ROS generation overwhelms the antioxidant defense mechanisms, leading to dramatic compromising of photosynthesis and the depletion of the energy supply [59, 60]. In this regard, the intimate interrelation between A/N-INV and reducing sugar content was assumed to regulate photosynthesis and control carbon metabolism in chloroplasts [23, 41, 61], and thus supply energy products to repair the damage [27, 28]. Interestingly, we found that *PtrA/NINV* localized to chloroplasts, implying that this protein may execute its function to provide sufficient reducing sugars to act in sustaining ROS homeostasis in this organelle [51]. In addition, INV was suggested to associate with hexokinase (HXK), which phosphorylates Glc to regulate ROS balance [18, 50, 59]. On the other hand, HXK-derived Glc-6-phosphate in the chloroplast is involved in the oxidative pentose phosphate pathway, which is an essential mechanism to control H_2_O_2_ scavenging [62, 63]. Accordingly, the transgenic plants experiencing abiotic stress accumulated significantly lower H_2_O_2_ relative to WT, suggesting that the chloroplasts in the transgenic lines may suffer from less serious oxidative damage, and thus exhibit better organelle viability.

In this study, the transgenic plants actively synthesized chlorophyll, which was verified by the higher chlorophyll content, more robust chlorophyll fluorescence imaging, and higher Fv/Fm in response to abiotic stress relative to WT. The correlation between the overexpression of A/N-INV and the higher chlorophyll levels is consistent with an earlier study, in which Arabidopsis CINV1 mutants displayed lighter green leaves compared with WT plants [23]. Similarly, the plastidic *At-A/N-Inv*E mutant exhibited inhibition of chlorophyll development due to the interruption of Suc hydrolysis and chlorophyll biosynthesis [22]. These studies, together with ours, implicate A/N-INV-mediated sugar metabolism in the modulation of oxidative stress and photosynthesis capacity. This interpretation is further supported by the analysis of the critical indices pertinent to photosynthesis, which showed that the transgenic lines exhibited higher Φ_PSII_, qP, and ETR, but lower NPQ compared to the WT. Based on our results it seems tempting to speculate that the transgenic lines might efficiently utilize the light absorbed by chlorophyll, triggering active photoprotection (qP) but compromised non-photochemical quenching (NPQ) [60, 64, 65]. Alternatively, the WT may absorb excessive light energy that exceeds its quenching capacity through photosystem II, leading to exacerbated damage to the photosynthetic apparatus. The maintenance of a robust photosynthetic capacity may represent one of the major physiological mechanisms explaining the enhanced abiotic stress tolerance of the transgenic lines overexpressing *PtrA/NINV* [5, 61, 66], congruent with the larger biomass in these lines.

## Conclusion

An alkaline/neutral invertase gene *PtrA/NINV* was isolated from trifoliate orange based on a previous study via SSH screening of a cold library. This gene is categorized as a stress-responsive gene because its expression was induced by multiple stresses. We observed that the protein encoded by *PtrA/NINV* was dual targeted to chloroplast and mitochondrion. Overexpressing *PtrA/NINV* in transgenic plants enhanced the degradation of Suc into reducing sugars, which exhibited enhanced tolerance to cold, salinity and drought stress. We ascribe the fortified stress tolerance to the diverse roles played by reducing sugars, such as roles in osmotic adjustment, more powerful ROS scavenging and more effective protection of the photosynthetic apparatus. Collectively, our data indicate that *PtrA/NINV* plays an important role in orchestrating sugar metabolism and stress responses and points to the great potential of *PtrA/NINV* for the genetic manipulation of crops for the purpose of enhanced stress tolerance.

## Methods

### Plant materials and multiple stress treatments

Two-month-old seedlings of trifoliate orange, grown in a greenhouse of National Center of Citrus Breeding, Huazhong Agricultural University, were used to analyze the expression of *PtrA/NINV*. The seedlings were washed and cultured for 3 days in water in a growth chamber (25 ± 2 °C, relative humidity 65 %, photoperiod 16 h light/8 h dark with a light intensity of 100 μmol m^−2^s^−1^) (unless otherwise stated, all chamber conditions are the same). For the cold and salt treatments, the seedlings were placed in a 4 °C incubator or immersed in 200 mM NaCl solution for 0, 6, 24, 72, and 144 h. For dehydration stress, the seedlings were placed on filter papers at ambient temperature for 0, 0.5, 1, 3, and 6 h. In addition, the seedlings were treated with ABA (100 μM), Suc (200 mM), or Glc (200 mM) for 0, 6, 24, and 48 h. Leaves were sampled at the designated time points, immediately frozen with liquid nitrogen, and placed at −80 °C until analyzed.

### Isolation of *PtrA/NINV* and gene sequence analysis

Total RNA was isolated from cold-treated leaves using RNAiso Plus RNA (TaKaRa, China). First strand cDNA was synthesized using RevertAid Reverse transcriptase (Thermo, USA) according to manufacturer’s instructions. We searched the database of *Citrus clementina* with the EST sequence and a gene GenBank file (accession no. XM_006419242.1) was retrieved, based on which a pair of specific primers (*PtrA/NINV*F/R, unless otherwise stated, all primers are listed in Additional file 4: Table S2) was designed to amplify full-length cDNA following standard procedures [67]. The amplified PCR product was cloned into the pMD18-T Vector (TaKaRa, China) and subsequently sequenced. The resultant sequence was then submitted to ORF finder server (http://www.ncbi.nlm.nih.gov/gorf/gorf.html) to identify the ORF, which was designated as *PtrA/NINV*. Molecular weight and theoretical *pI* of *PtrA/NINV* were calculated using Protparam. We used Motif scan and MemeV4.10.1 servers to identify the conserved domain, phosphorylation sites, and the distribution of motifs. The phylogenetic relationships between *PtrA/NINV* and the 57 protein sequences of alkaline/neutral invertase genes (accession numbers of the genes are shown in Additional file 5: Table S3) were analyzed in MEGA6 software using Neighbor-Joining dendrogram with 1000 bootstrap replicates and then visualized by FigTree v1.4.2. Multiple sequence alignment was performed with Clustal Omega and displayed by GeneDoc, while the gene structure display server was utilized to construct the *PtrA/NINV* gene structure. The prediction of targeting peptides at the N-terminus was done using Predotar v.1.03, WoLF SPORT, Mitoprot, TargetP, ChloroP [30–34], whilst the dual targeting was predicted by YLoc^+^[35].

### Quantitative real-time RT-PCR (qRT-PCR) analysis

The mRNA levels of *PtrA/NINV* in trifoliate orange and transgenic plants were determined by qRT-PCR. The extraction of the total RNA and the synthesis of the first strand cDNA were carried out as mentioned above. qRT-PCR was performed using Applied Biosystems® QuantStudio™ 7 Flex Real-Time PCR System (ABI, USA) and the SYBR® Green PCR kit (QIAGEN, USA) according to manufacturer’s instructions. Gene specific primers for qRT-PCR were used for the expression analysis, while *Actin* and *Ubiquitin* were used as internal reference genes for trifoliate orange and tobacco, respectively. Melting curves were performed after 45 cycles to verify primer specificity. Expression levels of *PtrA/NINV* in trifoliate orange at each time point were compared to initial treatment time, which was set as 1. In addition, the expression levels of *PtrA/NINV* in the transgenic plants were compared to WT (set as 1). Expression analysis for each time point or each treatment was conducted for at least two times, and representative data are shown as the mean values ± SE. The relative expression level was calculated using the 2^-∆∆CT^ method [10].

### Subcellular localization analysis of *PtrA/NINV*

The full-length ORF of the *PtrA/NINV* without stop codon was PCR-amplified with primers containing *Stu*I and *Mlu*I restriction sites. The PCR amplicon was ligated into the pMD18-T vector followed by the in-frame fusion to the 5´-end of GFP in the binary vector pCAMBIA 1302 under the control of the CaMV *35S* promoter. The constructs *PtrA/NINV::GFP* and GFP were independently transformed to *Agrobacterium tumefaciens* strain (GV3101). The epidermal cells of tobacco (*Nicotiana benthamiana*, seeds were provided by Prof. Feng Li of Huazhong Agricultural University) leaves were infiltrated with the bacterial suspension and incubated in the growth chamber for 2 days. To observe mitochondria, the leaves were immersed in 0.5 μM Mito Tracker® Red CMXRos (Meculare Probes Division, Invitrogen) for 15 min. GFP fluorescence and MitoTracker staining were detected with a confocal microscope (FV1000; Olympus, Tokyo, Japan) under green and red channels, respectively, whereas chloroplast autofluorescence was displayed using UV channel using Nikon microscope (Nikon, Japan).

### Generation of *PtrA/NINV-*overexpressing tobacco plants

*PtrA/NINV* ORF was acquired from the pMD18-T-*PtrA/NINV* vector by double digesting with *Sma*I and *Sac*I and ligated into the pBI121 plasmid, which uses the CaMV *35S* promoter to drive the expression of the *PtrA/NINV* ORF. *A. tumefaciencs* strain (GV3101) was transformed with this plasmid and was then used to transform tobacco (*N. nudicaulis*) [67], seeds of which were provided by Dr. Xuejun Chen (Yunnan Academy of Tobacco Agricultural Sciences, Yuxi, China). Transformants resistant to kanamycin (100 mg L^−1^) in the T_0_ generation were examined by PCR using the CaMV *35S* promoter-specific forward primer and *PtrA/NINV*-specific reverse primers (OE). Transcript levels of *PtrA/NINV* in the transgenic lines were determined by qRT-PCR as mentioned above. Three independent homozygous lines (#7, #8, and #39) in the T_3_ generation with higher *PtrA/NINV* expression levels were selected for further studies.

### Cold stress tolerance assay

Seeds of the transgenic and wild type plants were sown on MS medium [68] for one week and then transferred to soil pots. One week later, the seedlings were exposed to 4 °C for 72 h, and then exposed to a mild stress treatment at −1 °C for 24 h, followed by a recovery period at ambient environment for 3 days. The survival rates of tested plants were recorded and false color imaging was performed. In a separate experiment, 45-day-old plants were exposed to 4 °C for 72 h, and then exposed to lower temperature treatment at −1 °C for 48 h. The leaves were analyzed for the accumulation of ROS and electrolyte leakage, or immediately frozen in liquid nitrogen, and stored at −80 °C for further measurements.

### Salt stress tolerance assay

Seeds from WT and the transgenic lines were sterilized and germinated on MS medium for one week. The seedlings were transferred to fresh MS medium or MS containing 100 and 200 mM NaCl. The plates were vertically placed in a growth room, and the total root length was measured after 8 days of treatment. In another experiment, one-month-old transgenic and WT plants were grown in hydroponic solutions composed of either 25 % Hoagland solution or 25 % Hoagland solution and 150 mM NaCl. Two weeks later, plant growth was recorded and biomass (dry weight) of roots and aerial parts was measured. In addition, 300 mM NaCl was sprayed on the leaves of two-week-old seedlings for two weeks (twice a week), followed by spraying with water for another two weeks, and then survival rates were recorded. In parallel, 45-day-old potted plants of WT and transgenic lines were subjected to a salt treatment every four days, starting from 50 mM NaCl and reaching 300 mM NaCl, followed by observation of the phenotype and false colour imaging. The leaves were sampled for *in situ* ROS and cell death staining, or immediately frozen in liquid nitrogen, and stored at −80 °C for further measurements. Salt stress treatments using leaf disks were also performed. To this end, leaf discs of 1.5 cm in diameter were punched from one-month-old WT and transgenic plants. The leaf disks were floated on water or different concentrations of salt solutions (300 and 400 mM NaCl). After 72 h of treatment, the phenotype of leaf disks was photographed and the chlorophyll (*a*, *b*, and total) contents were measured.

### Drought stress tolerance assay

To assess drought tolerance, seeds of the transgenic lines and WT were sown in soil pots and the plants were grown for two weeks under a full watering regime. The plants were deprived of watering for two weeks, and then returned to regular irrigation for 3 days, and then the survival rates and chlorophyll fluorescence images were recorded. To estimate the water loss under dehydration conditions, leaves of two transgenic lines (#7, #8) and WT were detached and placed on filter papers at ambient environment for 2 h. The fresh weights of the leaves were examined every 15 min, and the rate of water loss was calculated by comparing with the initial weight. At the end of experiment, the leaves were collected for *in situ* ROS staining.

### Physiological and biochemical measurements

Electrolyte leakage (EL) was measured by investigating relative conductance as previously described [69]. To do this, the collected leaves were sliced and immersed in 15 ml of deionized distilled water, using a tube containing only the same volume of water as the control. Both tubes were shaken on a shaker (QB-206, Qilinbeier, China) for 1 h (20 rpm) at room temperature, then the initial conductivities of sample (C_1_) and blank (CK_1_) were measured by a conductivity meter (DSS-307, SPSIC, China). The tubes were boiled for 10 min and cooled down to room temperature, followed by measurement of the second conductivity (C_2_ and CK_2_). EL was represented by relative conductance (C) calculated using the following equation: C (%) = (C_1_ − CK_1_)/ (C_2_ − CK_2_) × 100. MDA content, H_2_O_2_ content, and the activities of antioxidant enzymes, including CAT (EC 1.11.1.6), SOD (EC 1.15.1.1) and POD (EC 1.11.1.7), were determined using the appropriate kits (Nanjing Jiancheng Bioengineering Institute, Nanjing, China) according to manufacturer’s instructions. Protein contents, when necessary, were colorimetrically quantified using Coomassie brilliant blue G-250 based on a previous method [70].

Suc content was measured with a specific kit (Nanjing Jiancheng Bioengineering Institute, Nanjing, China) according to manufacturer’s instructions. Reducing sugar content was measured using the DNS method [70] with slight modifications. To this end, 0.2 g of frozen sample was ground in 5 ml of ddH_2_O, and incubated at 30 °C for 20 min to extract the sugars. The extract was centrifuged at 4000 rpm for 5 min, and 1 mL of the supernatant was added to the DNS solution (1.3 M of preheated Na-K tartrate aqueous solution containing 6.3 g of 3,5-DNS, 262 mL of 2 M NaOH, 5 g of phenol, and 5 g of Na_2_SO_3_), followed by colorimetric reading at A_540_. The resulting values were compared with a calibration curve constructed using Glc.

To determine the A/N-INV activity, 0.2 g of sample was homogenized in 1 mL of cold extraction buffer (100 mM HEPES-KOH pH 7.4, 5 mM MgCl_2_, 1 mM EDTA, 1 mM EGTA, 1 mM PMSF, 5 mM DTT, 1 mL L^−1^ Triton X-100, 200 mL L^−1^ glycerol, and 5 mM thiourea) [71]. The extract was centrifuged at 14000 rpm, 4 °C for 15 min, and A/N-INV activity was evaluated in the supernatant that was added to total volume of 200 μL with 50 mM Bicine-KOH solution containing 0.1 M Suc (pH 7.6). The samples were incubated at 30 °C for one hour, and the DNS solution was directly added to measure reducing sugar content in the reaction mixture. Assays without incubation were used as a control. The A/N-INV activity was presented as μmol reducing sugar/mg protein/min.

Chlorophyll (*a*, *b*, and total) content was colorimetrically measured as previously described [72]. In addition, chlorophyll *a* fluorescence was recorded using an IMAGING-PAM chlorophyll fluorometer and Imaging WinGegE software (Walz, Effeltrich, Germany). For chlorophyll fluorescence imaging, the tested plants were illuminated under a single saturating pulse of >1800 μmol photons m^−2^ s^−1^. The dark–light induction curve (Kinetics) was performed according to a method carried out in [36] with slight alternations, based on which the operating quantum efficiency of the photochemistry (Φ_psII_), photochemical (qP) and non-photochemical (NPQ) quenching, and electron transfer rate (ETR) were acquired. The maximum quantum efficiency of the photochemistry (Fv/Fm) was obtained from first time point of Φ_psII_.

### *In situ* histochemical staining of ROS and cell death

*In situ* accumulation of O_2_^·-^ and H_2_O_2_ was detected by histochemical staining with nitro blue tetrazolium (NBT) and 3, 3’-diaminobenzidine (DAB), respectively [67]. Cell death was detected by staining with Evans blue (Sigma, USA), as previously described [73]. The stained leaves were bleached in 100 % ethanol at 75 °C for 15 min, and kept in 70 % ethanol prior to imaging.

### Statistical analysis

Each stress treatment was repeated at least twice. The abiotic treatments were repeated at least twice with three biological replicates for each line, and the results of a representative experiment are shown. The values presented are means ± SE, and analyzed via IBM SPSS Statistics statistical software (Version 19). Statistical differences were compared with a one-way analysis of variance (ANOVA) based on LSD’s multiple range test at significance level of *P* < 0.05 (*), *P* < 0.01 (**) and *P* < 0.001 (***).

### Availability of data and materials

The datasets supporting the conclusions of this article are included within the article and its additional files.

## Additional files

Additional file 1: Figure S1. Gene structure of *PtrA/NINV* and schematic distribution of the motifs in the A/N- INV protein sequences from different plants. A. Gene structure of *PtrA/NINV* based on the number and position of exons (brown box), introns (solid lines), and untranslated region (blue box). B. Motif distribution identified using MEME web server in *PtrA/NINV* (red box), and representative genes of group III (cytoplasmic) (blue box), group IV (mitochondrial) (yellow box), and group V (chloroplastic) (green box). (DOC 360 kb) Additional file 2: Figure S2. Multiple sequence alignment of glyco-hydro100 conserved domain from *PtrA/NINV* and representative genes of group III, IV, and V. Identical and highly conserved residues are shaded in dark or grey, respectively. The start and ends of glyco-hydro100 domain were shown by blue arrows. Phosphorylation sites are indicated with red arrows, while the catalytic residues are indicated with a yellow box. (DOC 1576 kb) Additional file 3: Table S1. Prediction analysis of subcellular localization of *PtrA/NINV* using different software (Doc). (DOC 32 kb) Additional file 4: Table S2. List of primer sequences used in this study (Doc). (DOC 29 kb) Additional file 5: Table S3. Accession numbers of the alkaline/neutral invertase genes used in this article (Doc). (DOC 68 kb)

## Abbreviations

A/N-INV: alkaline/neutral invertase
ABA: abscisic acid
Fru: fructose
GFP: green fluorescence protein
Glc: glucose
MDA: malondialdehyde
ROS: reactive oxygen species
Suc: Sucrose
WT: wild type
