# Supplementary figures and images for: PtrA/NINV, an alkaline/neutral invertase gene of Poncirus trifoliata, confers enhanced tolerance to multiple abiotic stresses by modulating ROS levels and maintaining photosynthetic efficiency
Source: BMC Plant Biol. 2016 Mar 29;16:76. doi: 10.1186/s12870-016-0761-0 (PMC4812658; doi:10.1186/s12870-016-0761-0)

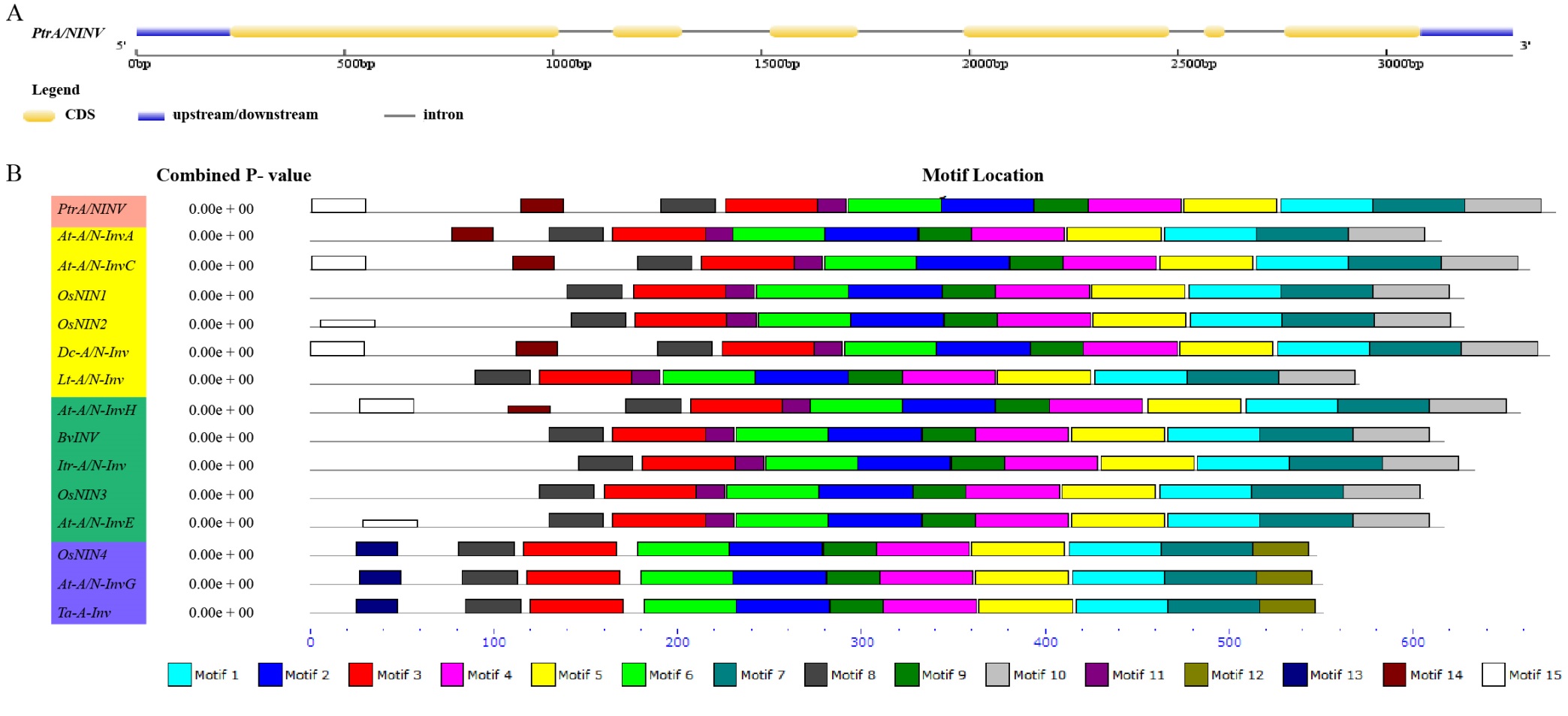


Figure S1

Supplement: Additional file 1: Figure S1. — Gene structure of PtrA/NINV and schematic distribution of the motifs in the A/N- INV protein sequences from different plants. A. Gene structure of PtrA/NINV based on the number and position of exons (brown box), introns (solid lines), and untranslated region (blue box). B. Motif distribution identified using MEME web server in PtrA/NINV (red box), and representative genes of group III (cytoplasmic) (blue box), group IV (mitochondrial) (yellow box), and group V (chloroplastic) (green box). (DOC 360 kb) [file 12870_2016_761_MOESM1_ESM.doc]

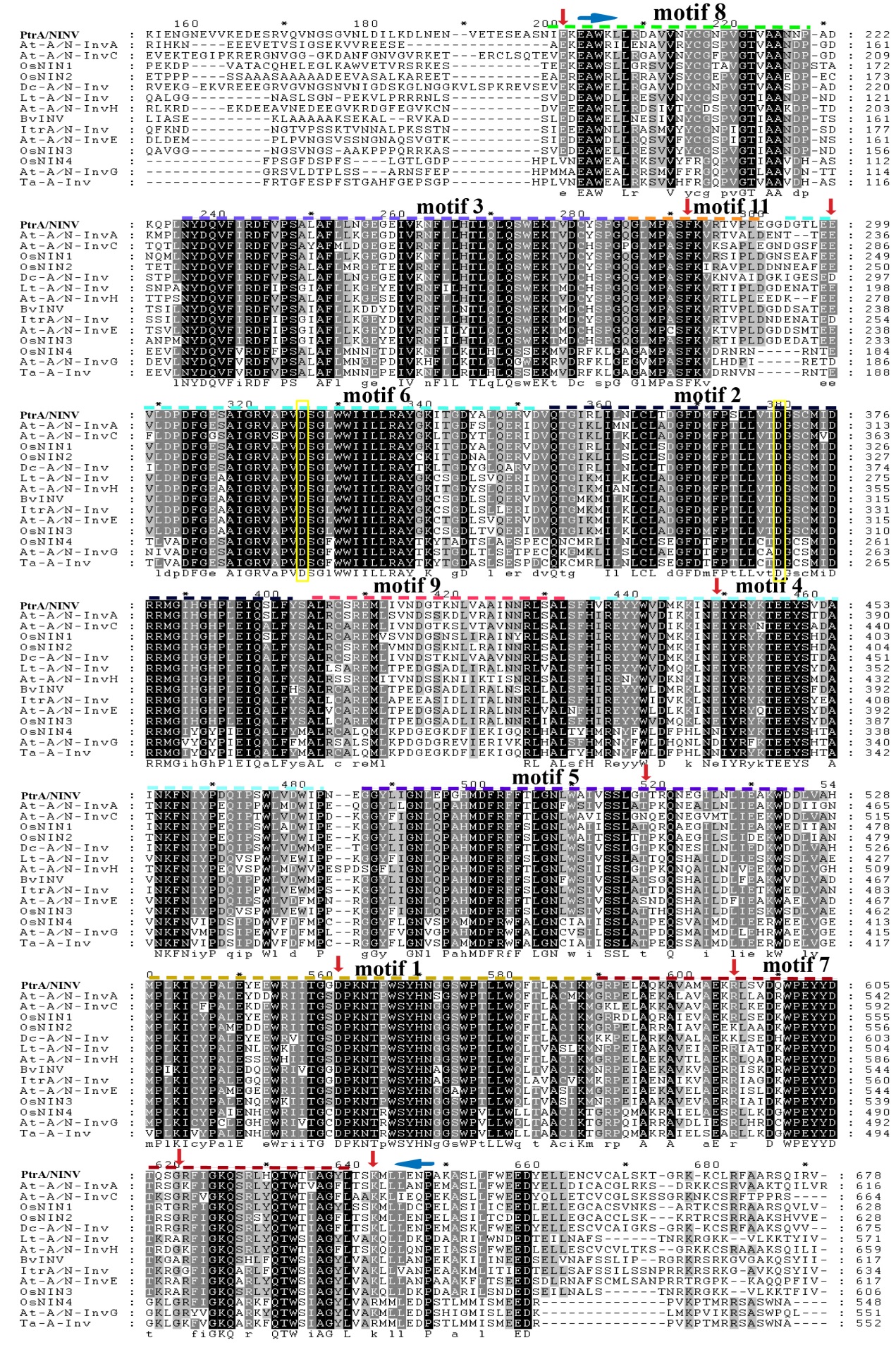


Figure S2

Supplement: Additional file 2: Figure S2. — Multiple sequence alignment of glyco-hydro100 conserved domain from PtrA/NINV and representative genes of group III, IV, and V. Identical and highly conserved residues are shaded in dark or grey, respectively. The start and ends of glyco-hydro100 domain were shown by blue arrows. Phosphorylation sites are indicated with red arrows, while the catalytic residues are indicated with a yellow box. (DOC 1576 kb) [file 12870_2016_761_MOESM2_ESM.doc]
