# Supplementary material for: PtrA/NINV, an alkaline/neutral invertase gene of Poncirus trifoliata, confers enhanced tolerance to multiple abiotic stresses by modulating ROS levels and maintaining photosynthetic efficiency
Source: BMC Plant Biol. 2016 Mar 29;16:76. doi: 10.1186/s12870-016-0761-0 (PMC4812658; doi:10.1186/s12870-016-0761-0)
Supplement: Additional file 3: Table S1. — Prediction analysis of subcellular localization of PtrA/NINV using different software (Doc). (DOC 32 kb) [file 12870_2016_761_MOESM3_ESM.doc]

**Table S1.** Prediction analysis of subcellular localization of *PtrA/NINV* using different softwares.

|  | Prediction tool | | | | | | |
| --- | --- | --- | --- | --- | --- | --- | --- |
|  | **Predotar** | **WoLF SPORT** | **Mitoprot** | **TargetP** | **ChloroP** | **^1^YLoc^+^ M1** | **^2^YLoc^+^ M2** |
| Mitochondria | 0.01 | 2 | 0.5940 | 0.047 | - | 98.01% | 82.23% |
| Chloroplast | 0.2 | 10 | - | 0.605 | 0.474 |  |  |
| ^3^Result | p | M or P |  | P | No chloroplast transit peptide | C | M and C |
| ^4^Cleaved sequence | **M**NTSSCIGIST**M**KPCCRILIGYRISSIFGVSHIRSNHKNVNNSSKLHSKSKLSCYNDAKCKVIGHKKGVIDLNRRAFFASGSNW | | | | | | |

**^1^Prediction result of** *PtrA/NINV* protein sequence **from first methionine residue.**

**^2^Prediction result of** *PtrA/NINV* protein sequence **from second methionine residue.**

^3^ p: Plastid, M: mitochondria, C chloroplast.

^4^Cleaved sequence **that** was predicted using **Mitoprot server.**
