# Supplementary material for: PtrA/NINV, an alkaline/neutral invertase gene of Poncirus trifoliata, confers enhanced tolerance to multiple abiotic stresses by modulating ROS levels and maintaining photosynthetic efficiency
Source: BMC Plant Biol. 2016 Mar 29;16:76. doi: 10.1186/s12870-016-0761-0 (PMC4812658; doi:10.1186/s12870-016-0761-0)
Supplement: Additional file 4: Table S2. — List of primer sequences used in this study (Doc). (DOC 29 kb) [file 12870_2016_761_MOESM4_ESM.doc]

**Table S2.** List of primer sequences used in this article

| **Gene** | **Primer sequences (5’-3’)** | |
| --- | --- | --- |
|  | **Forward primer** | **Reverse primer** |
| *PtrA/NINV* | TCCCCCGGGATGAATACTAGTAGCTGTATTGGAATCTC | CGAGCTCTTAGACACGGATCTGGGATCTTG |
| qRT-PCR | GGGAGGTTTATAGGCAAGC | ACGGATCTGGGATCTTGC |
| *Actin* | CCGACCGTATGAGCAAGGAAA | TTCCTGTGGACAATGGATGGA |
| *Ubiquitin* | GGTGTTTCCAGTGGCGGACG | TCCTCCCCTCAGCTACGGGGTAT |
| *PtrA/NINV::GFP* | AAGGCCTATG AATACTAGTAGCTGTATTGGAA | CGACGCGTGACACGGATCTGGGATCTTGCAGCA |
| OE | AAGATGCCTCTGCCGACAGTG | TTAGACACGGATCTGGGATCTTGCAG |
