# Supplementary material for: PtrA/NINV, an alkaline/neutral invertase gene of Poncirus trifoliata, confers enhanced tolerance to multiple abiotic stresses by modulating ROS levels and maintaining photosynthetic efficiency
Source: BMC Plant Biol. 2016 Mar 29;16:76. doi: 10.1186/s12870-016-0761-0 (PMC4812658; doi:10.1186/s12870-016-0761-0)
Supplement: Additional file 5: Table S3. — Accession numbers of the alkaline/neutral invertase genes used in this article (Doc). (DOC 68 kb) [file 12870_2016_761_MOESM5_ESM.doc]

**Table S3**. The accession number of the alkaline/neutral invertase genes used in this article

| Nom. | Gene ID | Organism | Accession number |
| --- | --- | --- | --- |
| 1 | *OsNIN1* | *Oryza sativa* | AK103334 |
| 2 | *OsNIN2* | *Oryza sativa* | AK120720 |
| 3 | *OsNIN3* | *Oryza sativa* | AK121301 |
| 4 | *OsNIN4* | *Oryza sativa* | AAX95795 |
| 5 | *OsNIN5* | *Oryza sativa* | NP_001052830 |
| 6 | *OsNIN6* | *Oryza sativa* | AK070884 |
| 7 | *OsNIN7* | *Oryza sativa* | AK065562 |
| 8 | *OsNIN8* | *Oryza sativa* | AK102741 |
| 9 | *MdoNIN1* | *Malusdomestica* | MDP0000163452 |
| 10 | *MdoNIN2* | *Malusdomestica* | MDP0000186866 |
| 11 | *MdoNIN3* | *Malusdomestica* | MDP0000146680 |
| 12 | *MdoNIN4* | *Malusdomestica* | MDP0000531557 |
| 13 | *MdoNIN5* | *Malusdomestica* | MDP0000261740 |
| 14 | *MdoNIN6* | *Malusdomestica* | MDP0000315220 |
| 15 | *MdoNIN7* | *Malusdomestica* | MDP0000297851 |
| 16 | *MDoNIN8* | *Malusdomestica* | MDP0000652278 |
| 17 | *MdoNIN9* | *Malusdomestica* | MDP0000095481 |
| 18 | *MdoNIN10* | *Malusdomestica* | MDP0000133399 |
| 19 | *MdoNIN11* | *Malusdomestica* | MDP0000319075 |
| 20 | *MdoNIN12* | *Malusdomestica* | MDP0000291284 |
| 21 | *At-A/N-InvA* | *Arabidopsis thaliana* | At1g56560 |
| 22 | *At-A/N-InvB* | *Arabidopsis thaliana* | At4g34860 |
| 23 | *At-A/N-InvC* | *Arabidopsis thaliana* | At4g06500 |
| 24 | *At-A/N-InvD* | *Arabidopsis thaliana* | At1g22650 |
| 25 | *AtA/N-InvE* | *Arabidopsis thaliana* | At5g22510 |
| 26 | *At-A/N-InvF* | *Arabidopsis thaliana* | At1g72000 |
| 27 | *At-A/N-InvG* | *Arabidopsis thaliana* | At1g35580 |
| 28 | *At-A/N-InvH* | *Arabidopsis thaliana* | At3g05820 |
| 29 | *At-A/N-InvI* | *Arabidopsis thaliana* | At4g09510 |
| 30 | *ItrA/N-Inv* | *Ipomoea trifida* | AAS79609 |
| 31 | *Sl-A/N-Inv* | *Solanumlycopesicum* | ABQ28669 |
| 332 | *Ta-A-Inv* | *Triticumaestivum* | AM295169 |
| 33 | *AnInvA* | *Nostoc sp. PCC 7120* | AJ491788 |
| 34 | *AninvB* | *Nostoc sp. PCC 7120* | AJ311089 |
| 35 | *NpInvA* | *Nostocpunctiforme* | AJ491790 |
| 36 | *NpInvB* | *Nostocpunctiforme* | AJ491789 |
| 37 | *PmMIT9313* | *Prochlorococcusmarinus MIT9313* | AJ491792 |
| 38 | *Pmpastoris* | *P. marinus subsp. Pastoris* | AJ491791 |
| 39 | *Pmmarinus* | *P. marinus subsp. Marinus* | NP_874763 |
| 40 | *Sm8102* | *Synechococcusmarinus WH8102* | AJ491793 |
| 41 | *Sy| WP_011243430* | *Synechococcus* | WP_011243430 |
| 42 | *Sy6803* | *Synechocystis sp. PCC6803* | CAD33848 |
| 43 | *BvINV* | *Beta vulgaris* | AJ422050 |
| 44 | *Dc-A/N-Inv* | *Daucuscarota* | Y16262 |
| 45 | *Lt-A/N-Inv* | *Loliumtemulentum* | AJ003114 |
| 46 | *Lcor-A/N-Inv* | *Lotus corniculatus* | AJ717412 |
| 47 | *MeNINV1* | *Manihotesculenta* | JN616390 |
| 48 | *MeNINV2* | *Manihotesculenta* | JQ339931 |
| 49 | *MeNINV3* | *Manihotesculenta* | JQ339932 |
| 50 | *MeNINV4* | *Manihotesculenta* | JQ782220 |
| 51 | *MeNINV5* | *Manihotesculenta* | KF533729 |
| 52 | *MeNINV6* | *Manihotesculenta* | KF533730 |
| 53 | *MeNINV7* | *Manihotesculenta* | JN801148 |
| 54 | *MeNINV8* | *Manihotesculenta* | JQ339933 |
| 55 | *MeNINV9* | *Manihotesculenta* | KF533731 |
| 56 | *MeNINV10* | *Manihotesculenta* | KF533732 |
| 57 | *CclA/NINV* | *Citrus clementina* | XM_006419242.1 |
